# Cryogenic study of the magnetic and thermal stability of retained austenite in nanostructured bainite

**DOI:** 10.1080/14686996.2019.1625722

**Published:** 2019-06-27

**Authors:** Arántzazu Argüelles, Florentina Barbés, Jose I. Espeso, Carlos Garcia-Mateo

**Affiliations:** a Dpto. de Ciencia de los Materiales e Ingeniería Metalúrgica, Edificio Departamental Este-Campus de Viesques-Universidad de Oviedo, Gijón, Spain; b Dpto. CITIMAC, Universidad de Cantabria, Santander, Spain; c MATERALIA Research Group, National Center for Metallurgical Research CENIM-CSIC, Madrid, Spain

**Keywords:** Bainitic steels, austenite-to-martensite phase transformation, nanostructured metals, magnetic properties, cryogenic temperature, 10 Engineering and Structural materials, 106 Metallic materials, 302 Crystallization / Heat treatment / Crystal growth, 500 Characterization, 503 TEM, STEM, SEM; 504 X-ray / Neutron diffraction and scattering, 600 Others

## Abstract

First magnetic characterization of a recently developed generation of carbide free bainitic steels, known as Nanobain, has been performed. Stability of its retained austenite at cryogenic temperatures has been studied by means of X-ray diffraction, microscopy, dilatometry and magnetic measurements. Two morphologies for this phase (*blocky*-type and *film*-type) appear in a different proportion depending on the chemical composition and the applied thermal treatment. Inhibition of the martensitic transformation, when decreasing the temperature down to −271°C, has been observed in those microstructures with higher proportion of *film-type* austenite. The paramagnetic state of austenite at room temperature seems to lead to different magnetic behaviors (ferromagnetic, antiferromagnetic) at cryogenic temperatures (T_C_ or T_N_ being around −23°C in all the studied samples), depending on the proportion of such morphological features. Furthermore, irreversibility with temperature on the evolution of such magnetic behaviors has been observed for all the studied bainitic structures and is proposed to be due to a magnetic proximity effect.

## GRAPHICAL ABSTRACT

**Figure:**
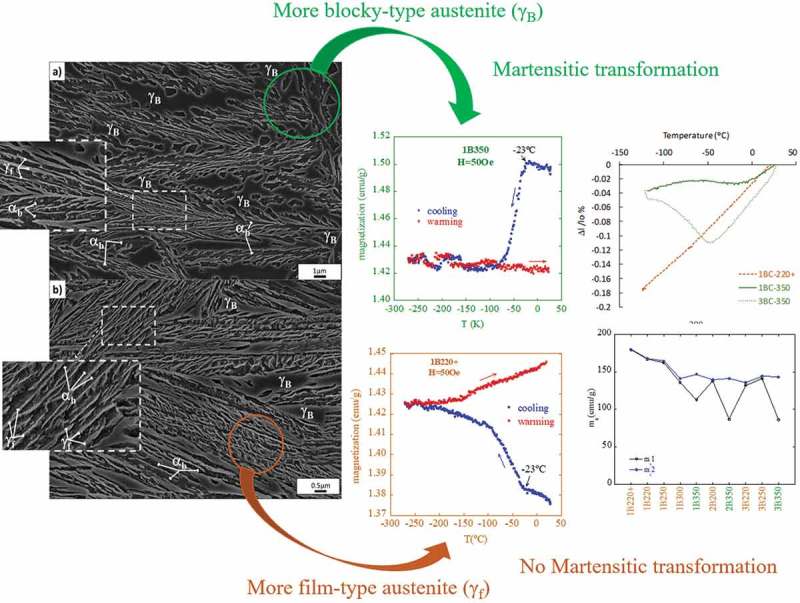

## 1. Introduction

Despite steel is a long standing material and object of a plentiful research from ancient times, it remains the object of cutting-edge studies nowadays. Its metallurgy has given rise to a wide range of mechanical properties that placed it among the most important industrial materials even at present [1]. However, this Fe-based alloy has much more to offer beyond its mechanical features. Thus, it is well known the main function of steel in rotating electrical machines [2] and in designing eddy-current and magnetic rail brakes for high-speed trains [3,4] relies on its electric and magnetic properties. Also recently, semimagnetic stainless steel has been tested and proposed as a suitable option in the design of high speed rotor permanent magnet machines [5] because it meets both the magnetic and the mechanical requirements, with a high yield strength. In this sense, the magnetic characterization of new developed steels is interesting by itself, as can provide useful information for potential applications.

In this work and for the first time, the new generation of steels, known as Nanobain, has been studied from a magnetic point of view [6]. Its microstructure, consisting of bainitic ferrite plates, with thickness around tenths of nanometers, alternating with carbon enriched retained austenite films, achieves exceptional mechanical properties (strengths in the range of 1.6–2.5 GPa and toughness of around 30 MPa$\sqrt{m}$ [7]) comparable to that of maraging steels, but with a lower production cost. The goal of the present work has been to get the first magnetic characterization of Nanobain steels, and more specifically, to find correlations between magnetic and microstructural features, which is hoped to help in designing the metallurgical scheme oriented to attain a particular magnetic behavior together with specific mechanical properties.

## 2. Materials and experimental methods

The chemical composition of the steels used in this work is listed in Table 1. All the alloys are high C steels with enough amount of Si (≥1.5%) to avoid cementite precipitation during the bainitic transformation [8]. Those alloys were selected for being able to transform into nanostructured bainite when isothermally transformed at low temperatures, see for example refs [6,9–12].

**Table 1.** Chemical composition of the analyzed alloys (wt.%).

Material	C	Si	Mn	Cr	Cu	Ni	Mo
Steel1	0.66	1.45	1.35	1.02	-	0.1	0.24
Steel2	0.98	2.90	0.77	0.45	0.21	0.16	-
Steel3	0.99	2.47	0.74	0.97	0.17	0.12	0.028

**Figure 1.**
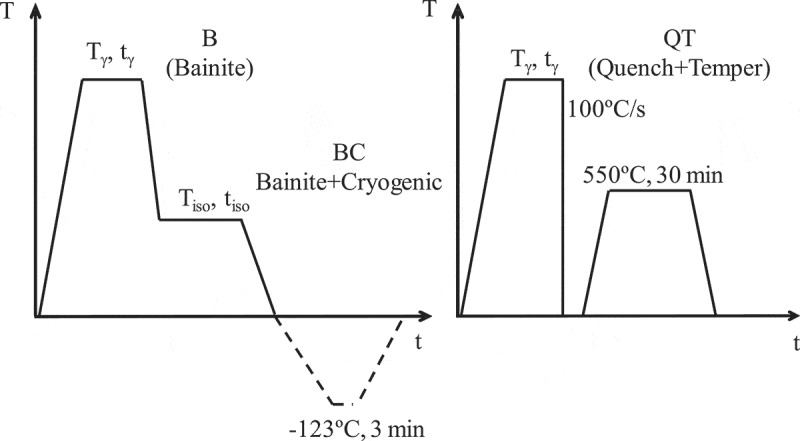
Heat treatment schemes; treatment B (Bainite) for obtaining the bainitic microstructures, treatment BC (Bainite+Cryogenic) cooling down after having obtained the bainitic microstructure, treatment QT (Quench+Temper) in order to obtain an austenite-free microstructure.

Through this work, different types of heat treatments were applied, whose purpose will become clear later when presenting the experimental results. In order to produce the desired bainitic structure, isothermal treatments after full austenitization were applied in conditions adapted to the different chemical compositions (treatment B in Figure 1). Selected bainitic microstructures were cooled down from room temperature (RT) to ~ −123°C and back to RT (treatment BC). Finally, with the purpose of generating a fully ferritic microstructure, samples of two of the alloys were quenched, creating a microstructure composed of martensite (α´) and retained austenite (γ), followed by a tempering treatment that allowed for the full decomposition of austenite into ferrite and scarce precipitates, (treatment QT in Figure 1).

Specifics of the different parameters used in the heat treatments are gathered in Table 2. It has to be highlighted that the austenitization and isothermal temperatures and times, as well as the cooling rates, were adapted to the different alloys according to previous experiences [6,9–12]. In order to easily identify the samples, the following labelling procedure has been used: a number identifying the steel category (as shown in Table 1), the acronym corresponding to the thermal treatment and the temperature of the isothermal treatment; the plus symbol in one of the experiments indicates extra time at the isothermal transformation temperature.

**Table 2.** Heat treatments parameters: T_γ_ and t_γ_ are the austenitization temperature and time respectively; T_iso_ and t_iso_ are the temperature and time respectively, for the formation of bainite by an isothermal treatment; T_cryo_ and t_cryo_ are the temperature and time, respectively, for the cryogenic treatment.

SAMPLE	T_γ_(°C)	t_γ_(min)	T_iso_(°C)	t_iso_(h)	T_cryo_(°C)	t_cryo_(min)
1B220+	900	5	220	168	-	-
1B220	900	5	220	24	-	-
1B250	900	5	250	8	-	-
1B300	900	5	300	5	-	-
1B350	900	5	350	4.5	-	-
1QT	900	5	-	-	-	-
1BC220+	900	5	220	168	−123	5
1BC350	900	5	350	4.5	−123	5
2B200	950	15	200	40	-	-
2B350	950	15	350	8	-	-
3B220	1050	5	220	40	-	-
3B250	1050	5	250	25	-	-
3B350	1050	5	350	7	-	-
3QT	1050	5	-	-	-	-
3BC350	1050	5	350	7	−123	5

While microstructures obtained with a heat treatment of the type B were all produced in the framework of industrial collaborations according to the procedures described in [13], heat treatments denoted as BC and QT were performed in a Bahr 805D high-resolution dilatometer (TA Instruments, USA) equipped with an induction heating coil. Helium was used as quenching gas and the temperature was controlled by a K type thermocouple welded to the central part of the sample surface. For the cryogenic treatment, He flowing through a cooling coil immersed in liquid N was used instead.

The length change associated with the different metallurgical events taking place during the heat treatments were recorded by a linear variable displacement transducer (LVDT) with a resolution of 0.05 μm. The mentioned treatments were performed using fused silica push-rods to measure longitudinal changes in length, given the small expansion coefficient of quartz (push rods), 0.5 × 10^−6^°C^−1^, when compared with the expansion coefficient of steel, approximately 10 × 10^−6^°C^−1^; it is safe to conclude that the contribution of the push rods to the measured change in length is negligible.

Quantitative X-ray diffraction (XRD) analysis was used to determine retained austenite and bainitic ferrite volume fractions. Samples were step-scanned in a Bruker AXS D8 X-ray diffractometer (BRUKER AXS, USA) with a rotating Co anode X-ray tube as a radiation source, Göebel mirror optics and a LynxEye Linear Position Sensitive Detector for ultra-fast XRD measurements. A current of 30 mA and a voltage of 40 kV were employed as tube settings. Operational conditions were selected to obtain X-ray diffraction data with a high signal to noise ratio. XRD data were collected during 2 hours over a 2θ range 35 − 135°, with a step size of 0.01°. In this study, the Rietveld analysis program TOPAS 4.2 (Bruker AXS) was used for quantification and calculation of the structural parameters of both, the retained austenite and the bainitic ferrite. Line broadening effects due to the lattice microstrains were analyzed with the double-Voigt approach [14]. In order to eliminate the instrumental contribution to peak broadening, instrument functions were empirically parameterized from the profile shape analysis of a corundum sample measured under the same conditions. The austenite carbon content was estimated using the well-known expression in ref [15], that relates the influence of different alloying elements to the lattice parameter.

XRD sample preparation was performed using standard metallographic procedures but introducing a final set of cycles of etching and polishing in order to remove the surface layer that has been plastically deformed during the grinding step. That surface layer may contain traces of martensite formed by transformation-induced plasticity (TRIP) of austenite due to sample preparation, which would underestimate its real fraction.

The microstructure was revealed after a standard metallographic preparation, followed by a final etching with a 2% nital solution. A JEOL JSM-6500 field emission gun scanning electron microscope (FEG-SEM; JEOL, Japan) operating at 10 kV was used to observe the microstructure.

Bainitic ferrite plate thickness (*t_α_*) was determined on SEM micrographs by measuring the shortest distance perpendicular to the longitudinal dimension of the ferrite plate, and correcting for stereological effects as described in reference [16].

The magnetic measurements were made with a Quantum Design Physical Property Measurement System (PPMS; Quantum Design, USA), equipped with a 9T superconducting magnet and able to cover a temperature range from −271 to 77°C (2 to 350 K, approximately). Saturation magnetization values were obtained from the hysteresis loop at RT of every sample resulting from treatments B and QT (m_s_1). Subsequently, a thermomagnetic analysis was performed between RT and −271°C (2 K) first cooling and then heating. After this analysis, a second value for the saturation magnetization (m_s_2) was obtained from the hysteresis loop at RT measured for each sample.

## 3. Results and discussion

### 3.1. Nanostructured bainite: microstructural characterization at room temperature

**Figure 2.**
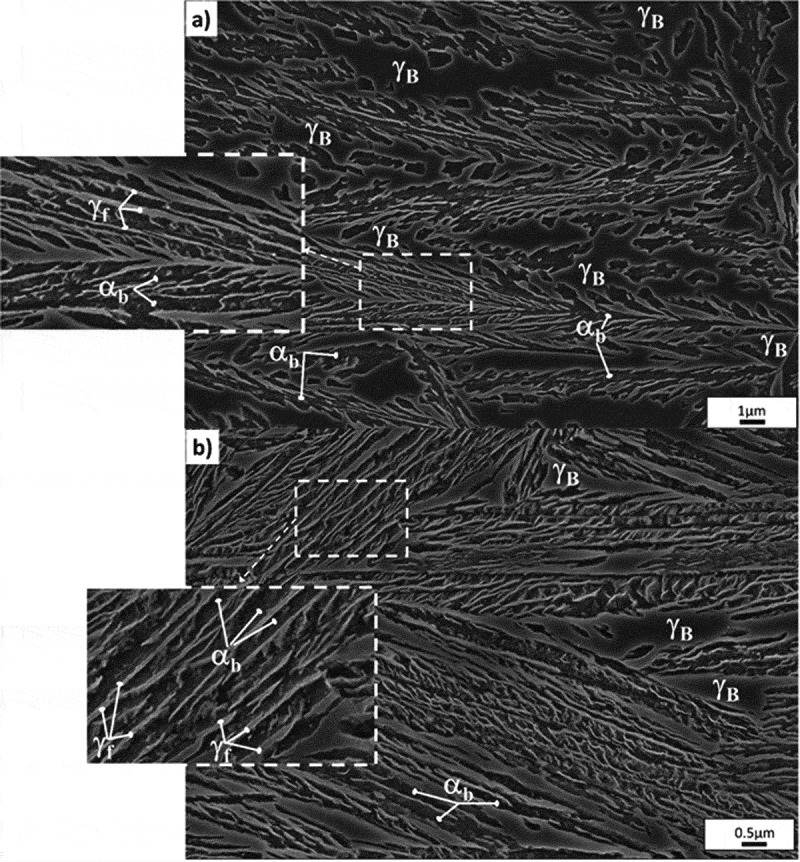
SEM micrographs showing examples of the bainitic microstructures of (a) 1B350, and (b) 1B220+, where bainitic ferrite (α_b_) and the two retained austenite morphologies, blocky (γ_B_) type and thin films (γ_f_) have been identified.

For all the steels under investigation, the microstructure after the B treatment consists of bainitic ferrite (α_b_) and retained austenite (γ). As reported in previous works, the microstructure is essentially carbide free and higher magnification techniques, such as transmission electron microscopy and atom probe tomography, only revealed scarce quantities of cementite precipitates [17,18]. Examples of the microstructures at selected temperatures can be found in Figure 2, corresponding to Steel1 after isothermal treatment at 350°C (sample 1B350) (Figure 2(a)) and at 220°C (sample 1B220+) (Figure 2(b)), respectively, where both phases have been identified. The darker long slender features are the plates of bainitic ferrite (α_b_), and the lighter phase found as *films* and more *blocky type* features correspond to retained austenite (γ). XRD results, presented in Table 3, corroborate that only these two phases are present, being the bainitic ferrite the predominant and the austenite the minor one. The maximum extent of transformation increases as transformation temperature decreases, i.e. bainitic ferrite fraction (V_αb_) decreases as isothermal temperature increases, which is expected from the incomplete reaction phenomena ruling the bainitic transformation [8]. Note that the time used in the isothermal treatment of the 3B220 sample did not allow a full bainitic transformation, so that more bainite could have been formed in a longer time, i.e. the final amount of bainite is smaller than that contained in the 3B250 sample.

**Table 3.** Chemical and microstructural parameters of the studied samples. V_γXRD_, V_αb_, V_α’_ are the volume fractions obtained by XRD for austenite, bainitic ferrite and martensite, respectively. V_γmagn_ is the volume fraction of austenite obtained by magnetic measurements. ε_γ_ is the microstrain and t_αb_ is the bainitic ferrite plate thickness. Those parameters for samples obtained after treatment B have been previously reported [6,9–11].

Sample	V_γXRD_ (%)±3%	V_αb_ (%)±3%	C_γ_ (wt.%) ±0.05	V_α´_ (%)±3%	V_γmagn_ (%)	ε_γ_ (%)	t_αb_ (nm)
1B220+	14	86	0.89	-	11	0.0041	38 ± 6
1B220	17	83	0.71	-	17	0.0036	42 ± 7
1B250	25	75	0.81	-	19	0.0032	42 ± 11
1B300	27	72	1.25	-	33	0.0027	61 ± 10
1B350	47	53	1.12	-	44	0.0018	79 ± 14
1QT	0	100					
1BC220+	12	88		-		0.0029	36 ± 4
1BC350	29	62		9		0.0020	-
2B200	29	71	1.19	-	28	0.0033	40 ± 11
2B350	57	43	1.34	-	54	0.0017	155 ± 30
3B220	41	59	1.21	-	31	0.0029	34 ± 8
3B250	23	77	1.43	-	26	0.0027	43 ± 10
3B350	45	54	1.32	-	55	0.0016	74 ± 13
3QT	0	96a					
3BC350	36	53		10.7		0.0023	-

^a^The 3QT sample contains 4% volume fraction of cementite.

The bainite transformation is a displacive and difussionless reaction, in which the ferrite is initially supersaturated with respect to carbon. The carbon excess in the bainitic ferrite is subsequently and rapidly partitioned into the residual austenite, but substitutional elements do not partition during the bainite reaction [8]. In the absence of carbide precipitation, prevented in the present steels by the use of silicon, the austenite carbon enrichment is such, at all transformation temperatures, that during the quenching to room temperature no martensite forms, i.e. the M_s_ (martensite start temperature) of C-enriched retained austenite is well below room temperature. Table 3 summarizes the results of the measured C content in austenite (C_γ_) where it is clear that the level of C enrichment is above of that of the bulk content.

Measurements of the bainitic ferrite plate thickness, t_αb_ (see Table 3), indicate a coarsening of the ferritic matrix as the transformation T increases (see Figure 2). These results are in agreement to what is expected and already extensively reported, see for instance refs [16,19,20]. In this kind of microstructures, austenite exhibits a well known multi-scale character associated with two distinguishable morphologies, i.e., thin films (γ_f_) between platelets of bainitic ferrite and blocks between sheaves of bainite (γ_B_), see for example ref [9,10,21–23]. Those have identified in Figure 2, i.e. in the main micrograph the *blocky-type*, and the *film-type*, due to their much smaller size, are identified in the higher magnification inset. The two different morphologies are well known for also having very different carbon contents in solid solution, i.e. the thin films are far richer in carbon than the blocks [23–29].

**Figure 3.**
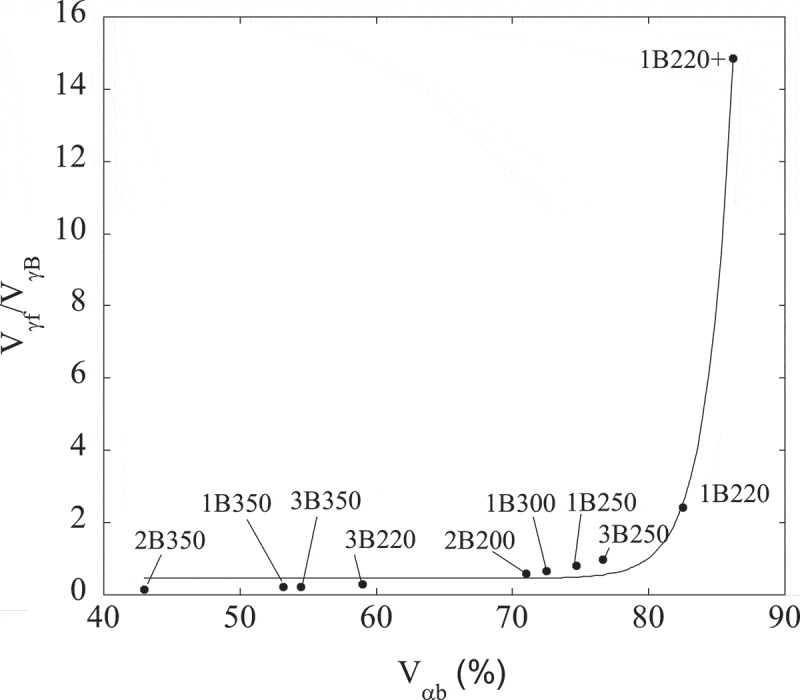
Relationship between the $V_{\gamma f}/V_{\gamma B}$ ratio (thin film/blocky type austenite) and the amount of bainitic ferrite (V_αb_) present in the studied microstructures. The line is just a guide for the eye.

There are two important effects on the microstructure as a consequence of decreasing the transformation temperature. On the one hand, as the fraction of bainitic ferrite increases, the fraction of thin *films* of austenite (V_γf_) increases at the expense of that of *blocky* austenite (V_γB_). An estimate of the ratio between both morphologies can be done following the procedure described in ref [30], where it is assumed that about 15% of the volume contained within the boundaries of a bainite sheaf consists of retained austenite films interspersed with bainite plates. Results obtained in this way are summarized in Figure 3, presented as a function of V_αb_.

The largest fraction of film type retained austenite is obtained for the sample 1B220+, while samples treated at the highest austempering temperature have the largest fraction of *blocky* type austenite (1B350, 2B350, 3B350). These results are consistent with what is observed in the micrographs shown in Figure 2, and they are expected from the incomplete reaction phenomena [8].

The displacive character of the bainitic transformation implies that there is a shape change along the transformation. The plastic relaxation of this shape change commonly takes place via generation of both, dislocations in the austenite/bainitic ferrite interface, and also micro/nano-twins in the austenite, in contact with bainitic ferrite plates [31–33]. As the transformation temperature decreases, there is an increase in the dislocation density caused by the yielding of the austenite. If we consider that microstrain is directly related to the dislocation density [34–36] (see Table 3) it is clear that indeed, the lower is the transformation temperature, the higher is the dislocation density introduced in the microstructure. Note that the differences in microstrain between 1B220+ and 1B220 correspond to a restoration process due to the extended heat treatment [37].

### 3.2. Nanostructured bainite: a preliminary magnetic characterization at room temperature (M versus H)

In order to get an initial insight into the magnetic behavior of these bainitic microstructures, their hysteresis loops at room temperature (RT) have been measured.

**Figure 4.**
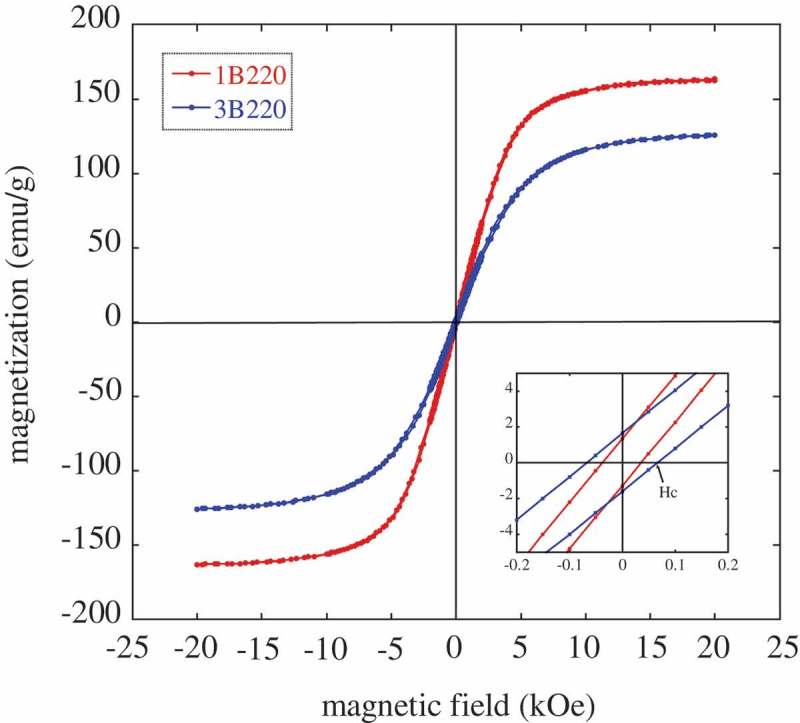
Measured magnetization as a function of the applied magnetic field (hysteresis loops) of the 1B220 and 3B220 samples at RT. The inset shows the detail around 0 Oe in order to better appreciate the coercive field, H_c._

An example of the curves thus obtained is presented in Figure 4 for the 1B220 and 3B220 microstructures. From such curves, the value of the coercive field (H_C_) is obtained, and gathered in Table 4. The obtained values range between 29–67 Oe, close to the coercitivities corresponding to other steel grades [38–40], and closer to those values of soft magnetic materials than to those of hard magnetic ones, as is well known Hc is typically less than 10 Oe for the first ones and more than 100 for the second ones [41].

**Table 4.** Magnetic parameters obtained for the studied samples: coercitivity (H_c_), saturation magnetization for the bainitic microstructures (m_s_1), and that of the same microstructures after undergoing a thermal cycle cooling down to −271°C and back to room temperature (m_s_2).

Sample	H_c_(Oe)	m_s_1(emu/g)	m_s_2(emu/g)
1B220+	29	179.6	179.9
1B220	37	166.5	167.9
1B250	42	162.4	164.4
1B300	60	135.6	141.3
1B350	40	113.2	146.8
1QT		191.1	
2B200	57	137.8	139.8
2B350	38	86.9	141.2
3B220	67	131.9	135.7
3B250	67	140.9	144.4
3B350	65	86.1	143.2
3QT		186.7 (189.13a)	

^a^Value corrected after considering the contribution of the cementite present in the microstructure, as explained in the text.

**Figure 5.**
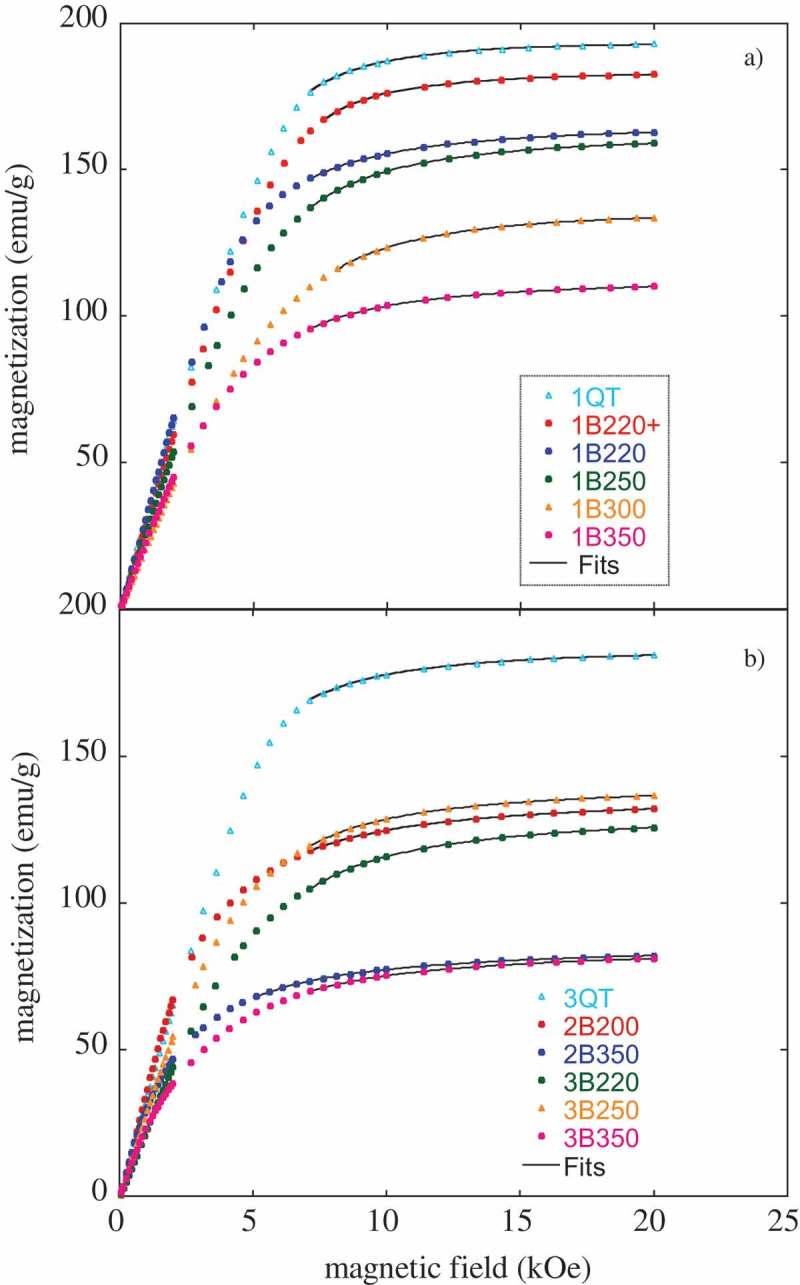
Measured magnetization as a function of the applied magnetic field, at RT, for (a) samples 1B, (b) samples 2B and 3B. The lines correspond to fits with Equation (2).

The initial magnetization curves, at RT, are presented in Figure 5 for all the studied samples. It is well known that ferromagnetic materials follow the so-called law of approach to saturation given by Equation (2), where m_s_ is the saturation magnetization and the parameters *a* (in Oe) and *b* (in Oe^2^) are positive and related to crystallographic anisotropy and internal strain [42]. As previously reported by other authors, the saturation magnetization value in steel grades can be obtained from a fit of the experimental data to Equation (2) [43,44]. Such these fits, for an applied field H in the range 7000 Oe < H < 20,000 Oe, are presented in Figure 5. They result in the m_s_ values denoted as m_s_1 in Table 4.

(2) $$m=m_{s}\left(1-\frac{a}{H}-\frac{b}{H^{2}}\right)$$

All the values obtained for the saturation magnetization are lower than that of pure Fe (217 emu/g) [45] and close to values reported for other steel grades [43,46–49]. Differences between them can be attributed to the chemical composition and microstructural differences. It can be seen, as expected from references [43,44,46,48,50,51], that for the same composition there is a strong correlation between the amount of ferritic phase and the m_s_ values, i.e. the highest values corresponding to the QT samples, which are almost 100% ferritic (Tables 3 and 4), are consistent with the absence of austenite (paramagnetic at RT) in the microstructure. Thus, a continuous decrease of m_s_ value with increasing austempering temperature (T_iso_) is observed for the three analyzed steel grades.

The mentioned strong correlation between the m_s_ values and the fraction of ferritic (ferromagnetic) phase has been exploited as a way for obtaining an estimate of the paramagnetic phase (austenite) present in the microstructure of steels (see, for example, [43,44,46,48,50,51]). Thus, the volume fraction of the retained austenite can be determined from the saturation magnetization value according to Equation (3) [43,44],

(3) $$V_{\gamma magn}=100\left(1-\frac{m_{s}}{m_{s}\left(i\right)}\right)$$

where m_s_ is the saturation magnetization of each sample and m_s_(i) is the intrinsic saturation magnetization, corresponding to an austenite-free microstructure with the same chemical composition. The sample 3QT contains, besides ferrite, also a 4% volume fraction of cementite, so in order to correct the experimental m_s_ value (~187 emu/g) a lever rule was applied considering that cementite saturation magnetization is ~129 emu/g [52,53]. The resulting corrected value for m_s_ is ~190 emu/g, which is close to that obtained in sample 1QT.

**Figure 6.**
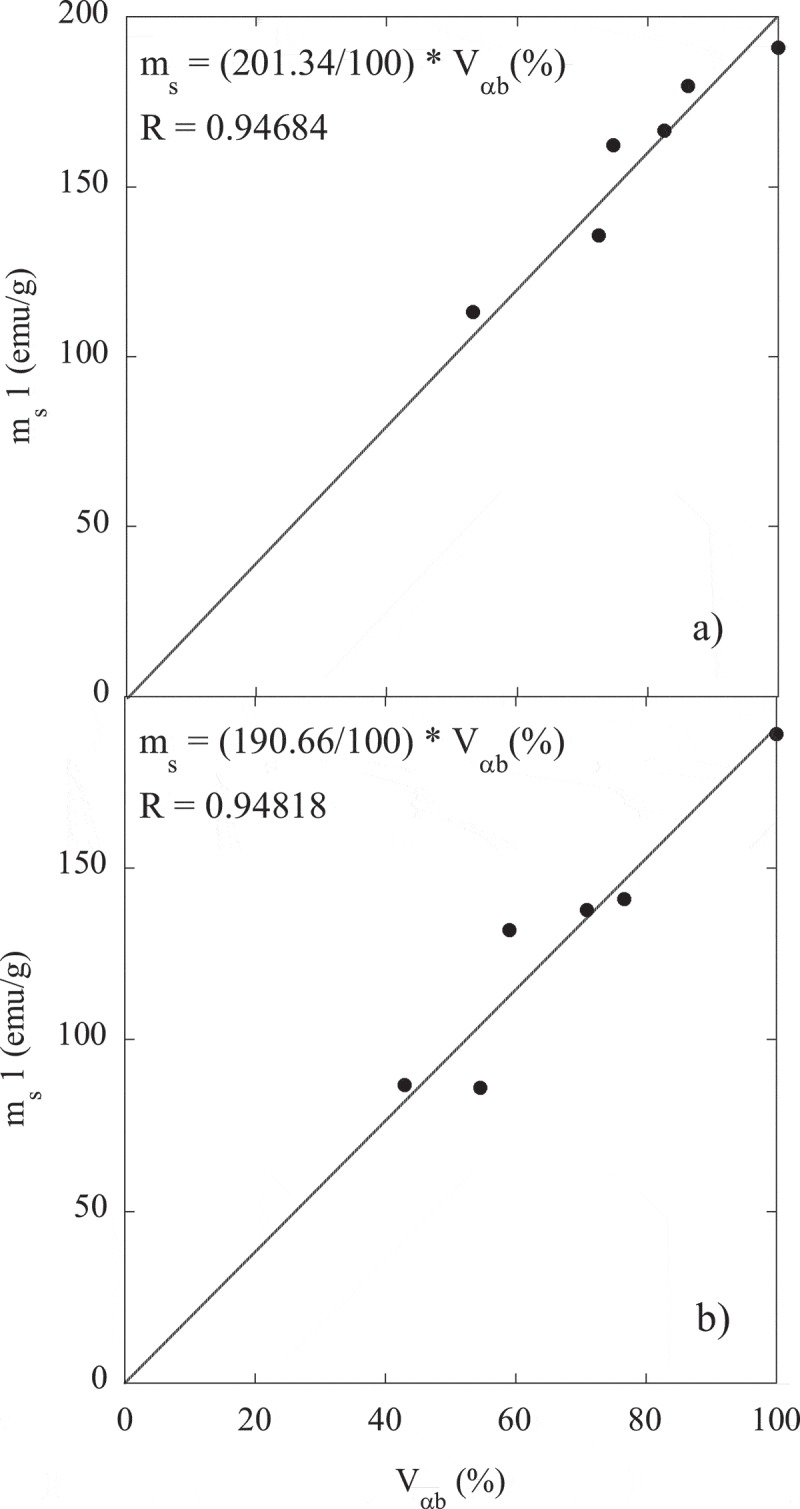
Saturation magnetization values, m_s_1, versus ferrite fraction (V_αb_) for (a) Steel1; (b) Steel2 and Steel3. The lines correspond to linear fits passing through the origin. The error associated to m_s_1 is about 0.15%, and for V_αb_ is ±3%.

**Figure 7.**
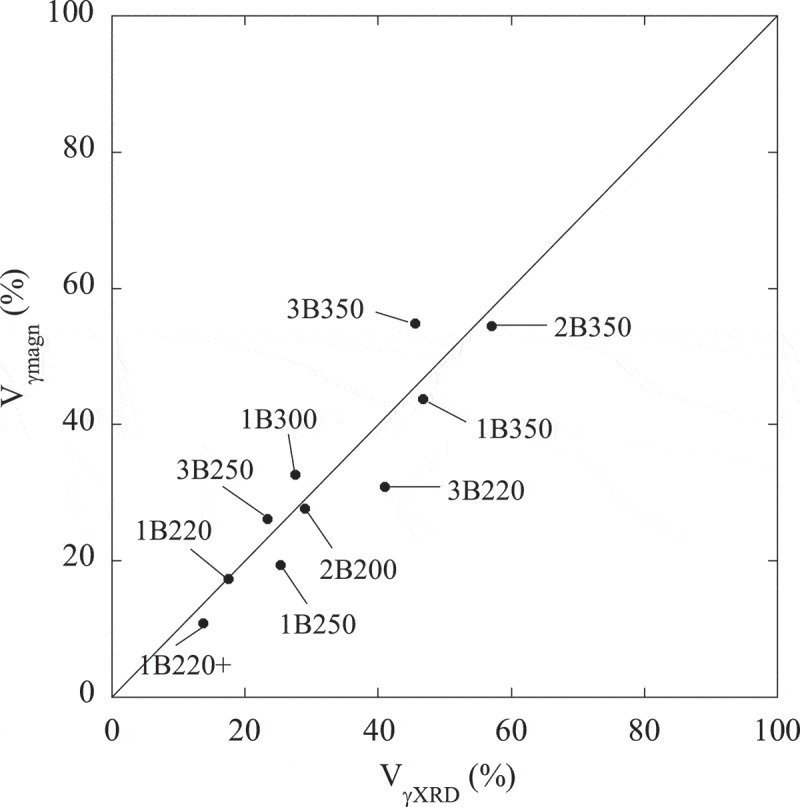
Austenite volume fraction obtained from two different methods: magnetic measurements (V_γmagn_) and XRD (V_γXRD_). The error associated to V_γXRD_ and V_γmagn_ is ±3% and ± 0.2% respectively.

Furthermore, by plotting the measured volume fraction of bainitic ferrite, V_αb_, presented in Table 3, against the specific saturation magnetization, m_s_1, reported in Table 4, it is possible to perform a linear fit, forcing it to pass through zero, as m_s_ is supposed to be zero for a fully austenitic microstructure. From these fits, the intrinsic m_s_(i) yields a value of ~201 emu/g for Steel1 (see Figure 6(a)), and a value of ~191 emu/g for Steel2 and Steel3 (see Figure 6(b)). Such obtained values show a reasonable level of agreement with those for the corresponding QT samples (see Table 4). Despite small chemical composition differences between Steel2 and Steel3, both steel grades have been treated together as it was checked that independent fits lead to similar values for m_s_(i). For the sake of clarity, a comparison of the austenite content obtained from both methods, XRD and magnetic measurements (considering m_s_(i) values from fits), is also presented in Figure 7. It can be seen that the values obtained by both methods are in good agreement, differing for most of the samples in no more than 6%, according with results reported by other authors in different steel grades [44,50].

### 3.3. Thermomagnetic measurements (M versus T)

In order to obtain further information on the austenite present in these samples, thermomagnetic curves have been measured for each one of them, decreasing the temperature down to −271°C and increasing it up again to 27°C, while a small and constant magnetic field of 50 Oe was applied. Taking into account that ferrite gets ferromagnetically ordered below ~770°C, and cementite below ~207°C, it can be assumed that any anomaly at lower temperatures has to be related to either the austenite magnetic behavior or to structural transformations from such phase.

**Figure 8.**
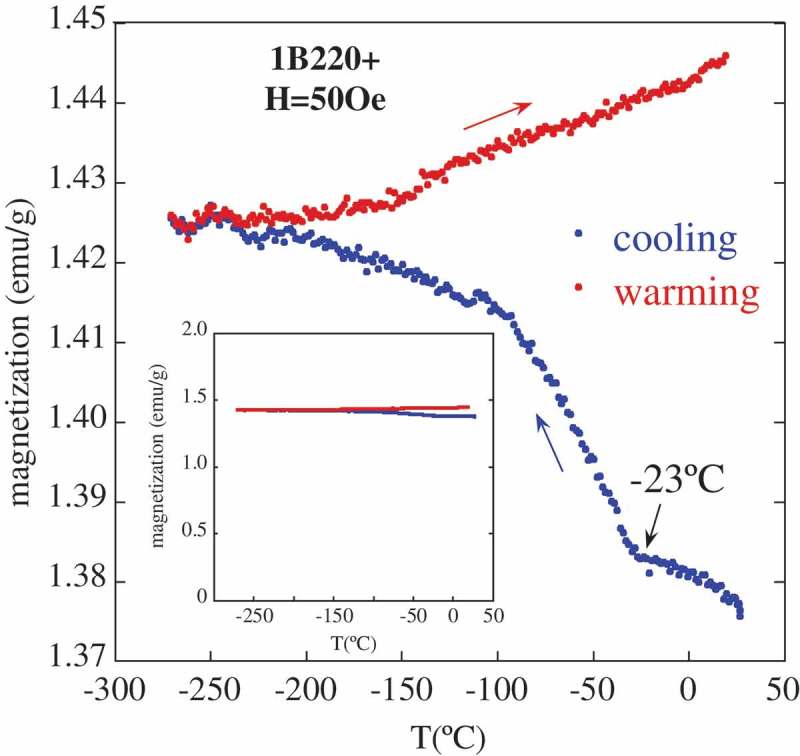
Thermomagnetic curve for 1B220+, measured during cooling from RT down to −271°C and heating back to RT. The inset shows the same curve at a different scale to realize about the relative change in the magnetization.

Some of the studied samples, with a high fraction of bainitic ferrite, and therefore a high fraction of thin films of austenite over the *blocky-type* (Figure 3), show a behavior in the M(T) curve such as that of 1B220+ (see Figure 8) with a sharp increase in their magnetization when decreasing T down from ~ −23°C. This increase constitutes around 3% of the total magnetization value at this temperature range, what means a small variation respect to the total magnetization value of the ferromagnetic iron phase (see inset in Figure 8). The significance of this transition will be clear later in the text.

**Figure 9.**
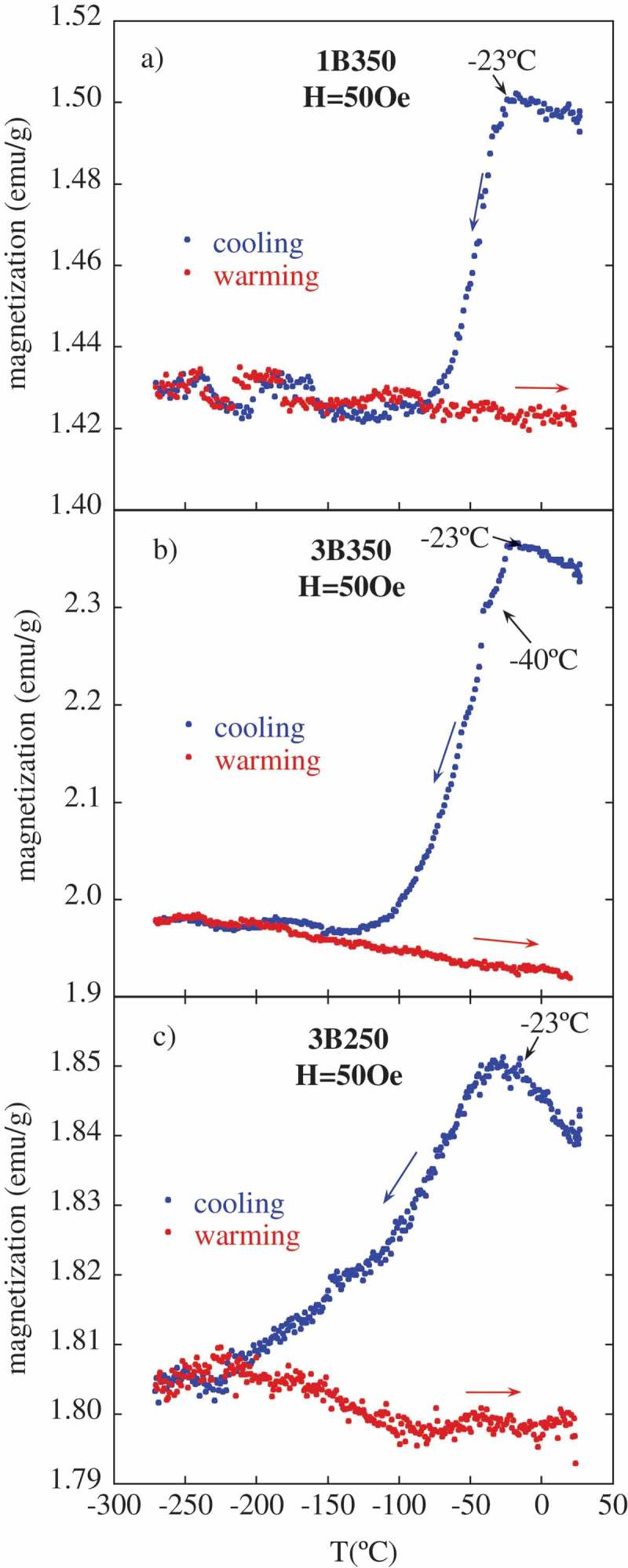
Thermomagnetic curves for (a) 1B350 sample; (b) 3B350 sample; (c) 3B250, measured during cooling from RT down to −271°C and heating back to RT.

On the contrary, magnetization decreases abruptly when decreasing T to values lower than that value of ~ −23°C in samples with a bigger amount of retained austenite, mainly of *blocky-type*, such as 1B350 and 3B350 (see Figure 9(a,b), respectively). Finally, a third behavior, intermediate between those both already mentioned, is exhibited by other samples, such as 3B250 (see Figure 9(c)) or 1B250, with a ‘cusp’ centered around the same temperature (~ −23°C). Such samples contain a similar amount of both *film-type* and *blocky-type* austenite (see Figure 3).

Furthermore, it is worth to highlight that, for all the studied samples, irreversibility is observed for the magnetization evolution when returning to RT after cooling (see Figures 8 and 9), which will be discussed later.

### 3.4. Stability of retained austenite in the cryogenic regime

**Figure 10.**
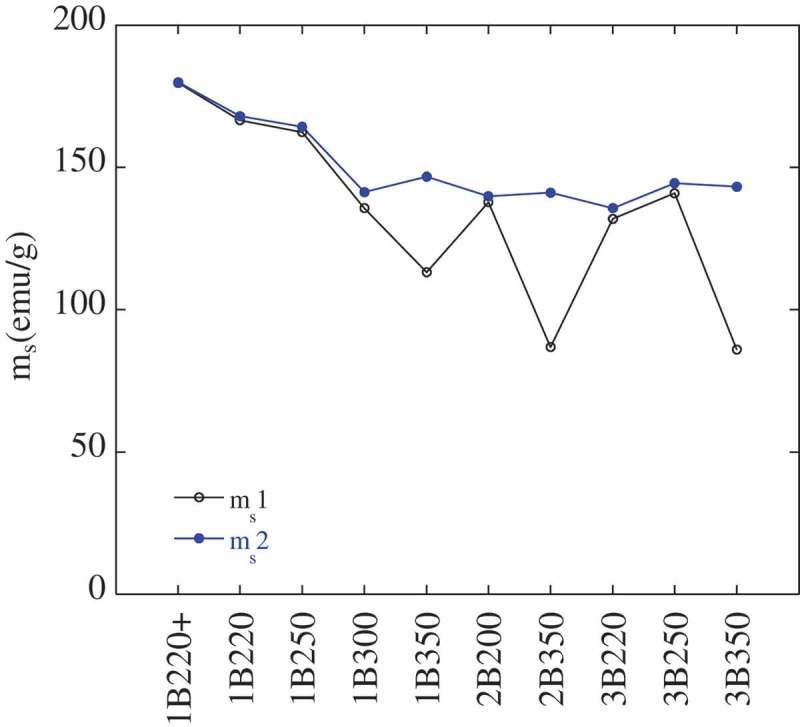
Comparison of the saturation magnetization values before (m_s_1) and after (m_s_2) performing the thermomagnetic measurement, i.e. cooling down to −271°C. The error associated to m_s_ is about 0.15%.

As thermomagnetic curves suggest changes of either structural or magnetic nature, a second hysteresis loop has been measured at RT, for every sample, after cooling down to −271°C. This second measurement of the hysteresis loops let us check if there are differences in the new saturation magnetization value (m_s_2) respect to that obtained from the virgin microstructure (m_s_1). These results are presented in Table 4 and, for discussion purposes, are plotted in Figure 10.

**Figure 11.**
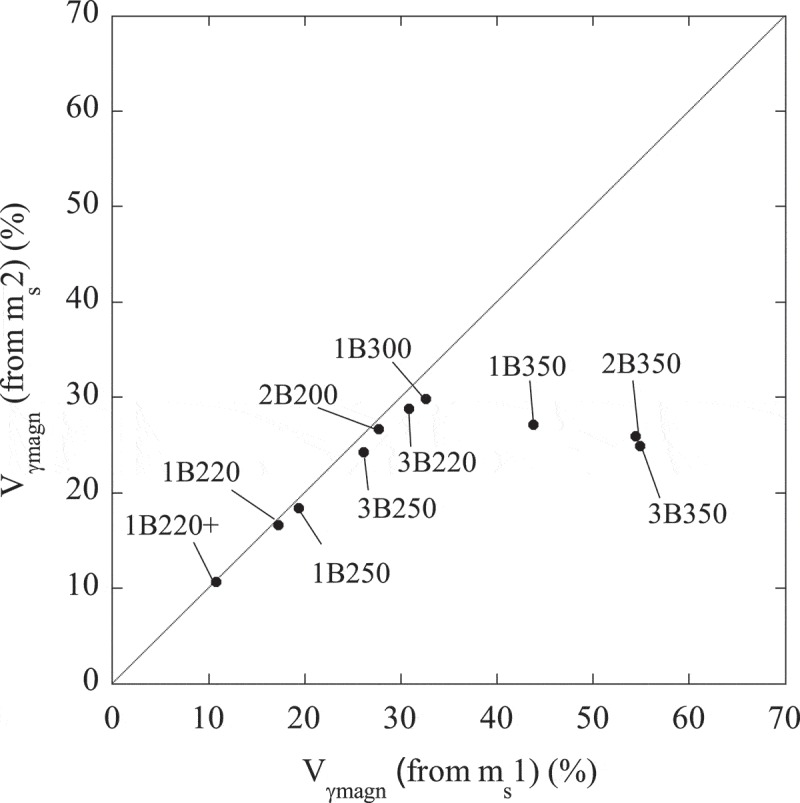
Correlation between the volume fraction of austenite (V_γmagn_) obtained from m_s_1, and m_s_2. The associated error is about 0.2%.

The higher value of m_s_2 respect to m_s_1 for the samples 1B350, 2B350 and 3B350 is indicative of a higher fraction of a ferromagnetic phase, i.e. partial transformation from austenite (paramagnetic at RT) to martensite (ferromagnetic at RT), which is very likely to occur when cooling down to −271°C [46,54–56]. On the contrary, in those samples where the m_s_1 and m_s_2 values are similar (see for example 1B220+), the occurrence of such transformation is unlikely and the microstructure is expected to be stable during the whole thermal cycle. A more direct way to visualize this is by means of Equation (3), from which V_γmagn_(1) was calculated. Using this expression, new V_γmagn_(2) values have been obtained from m_s_2 (see Table 4) and they are plotted versus the previous V_γmagn_(1) ones in Figure 11. According to these results, the microstructures with the lowest V_γf_/V_γB_ ratio, i.e. with higher fractions of *blocky* austenite (see Figure 3), as those transformed at 350°C, exhibit the highest degree of austenite transformation during the cryogenic treatment. Note that these samples are the ones that deviate from the perfect correlation line.

Before going deeper in the discussion, it is essential to comment on the parameters that may affect the thermal stability of austenite against martensitic transformation.

Martensitic transformation is displacive, involving the coordinated movement of atoms; and since the glissile transformation interface has a dislocation structure that has to move through any obstacles that exist in the austenite, martensitic transformation cannot be sustained against strong defects such as grain boundaries. Less drastic defects, such as dislocations, also hinder the progress of such transformations, but can often be incorporated into the martensite lattice [57].

The resistance of austenite against the martensitic transformation partially depends on its chemical composition, and elements such as C, Mn, Si and Al [22,58,59] significantly enhance the austenite stability; among them C is the element that exhibits the strongest influence. As already mentioned, the two different morphologies of retained austenite are strongly linked to different carbon contents, i.e. the thin films are far richer in carbon than the blocks. Since carbon is an interstitial solute, such differences also imply big differences in their thermal stability [60].

The differences in size of the austenitic features also have an important effect per-se on its stability against martensitic transformation [61–64]. This is in part due to the fact that small retained austenite islands contain lower potential nucleation sites for the transformation to martensite and, consequently, require a greater total driving force for the nucleation of martensite [65]. Therefore, the smallest austenitic features are more stable than the largest ones because of both their size/morphology and their carbon content.

Then there is the influence that the strength of the surrounding matrix, bainitic ferrite, has on the stability of the retained austenite. Theoretically, the refinement of the bainitic ferrite plates with different crystallographic orientations increases the stability of the retained austenite. If the latter is closely surrounded by the relatively rigid and refined bainitic ferrite, the stability of the retained austenite also increases due to the geometrical restrictions imposed by the surrounding bainitic ferrite laths [66,67]; in other words a stronger matrix may prevent the martensitic transformation.

Thus, in this work it is proposed a microstructural stability parameter (*msp*) that accounts for almost all the mentioned factors influencing the stability of austenite, i.e. carbon content (C_γ_), dislocation density (microstrain ε_γ_), morphology (the ratio r = V_γf_/V_γB_) and strength of the matrix (plate thickness t_αb_). Such parameter is therefore defined as being proportional to

(4) $$msp\;\;∼\;C_{\gamma }r\varepsilon_{\gamma }/t_{\alpha_{b}}$$

**Figure 12.**
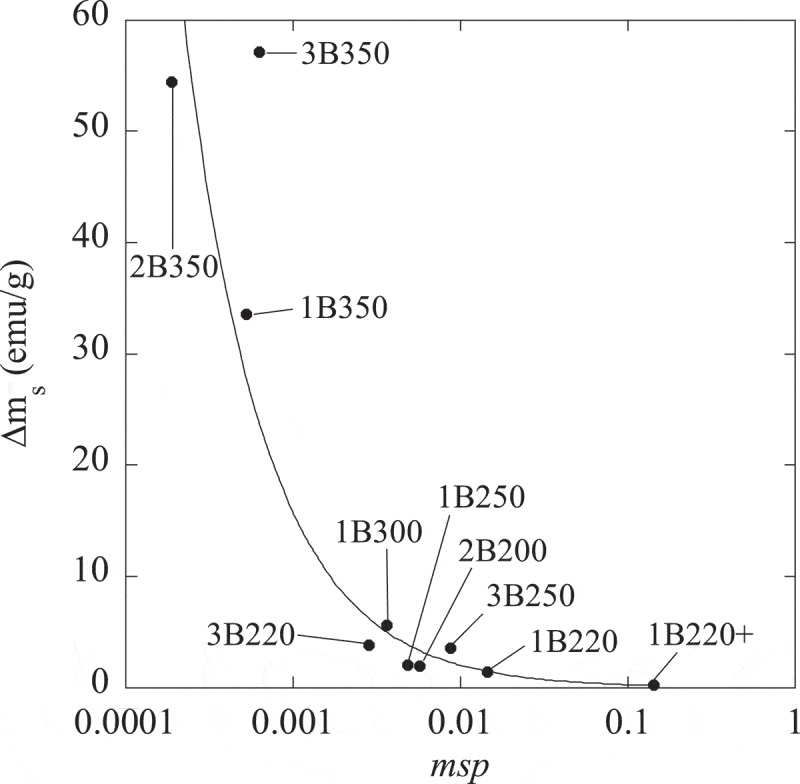
Microstructural and magnetic stability parameters, *msp* and Δm_s_ respectively, as defined in the main body of the text. Note that, for the sake of clarity, the microstructural parameter has been represented in log scale. The solid line is just a guide for the eye.

The exact values of the different parameters can be found in Table 3 and Figure 3. The higher the value of this product is, the more stable is the austenite within the initial bainitic microstructure (before the cryogenic treatment) against martensitic transformation. This fact is strongly correlated with the variation of m_s_ when cooling sample down to −271°C, as it is shown in Figure 12.

In this sense, and from the magnetic point of view, an equivalent stability parameter could be defined as Δm_s_ = |m_s_1-m_s_2|, since, as already discussed, a zero or very small value for this parameter is indicative of the austenite stability (paramagnetic at RT) respect to its transformation in martensite (ferromagnetic at RT) when undergoing the cryogenic treatment.

To further corroborate these results and the present discussion, and by means of dilatometry, a thermal cycle in the cryogenic regime (BC treatment in Figure 1) has been applied in three different microstructures: one where the austenite is considered as very stable (1B220+ sample), and other two where, according to the data so far presented, some degree of austenite transformation has occurred, i.e. 1B350 and 3B350 microstructures. The new samples were codified as 1BC220+, 1BC350 and 3BC350. Due to the equipment technical specifications, the minimum temperature is −123°C (the exact parameters of the thermal cycle are shown in Table 2). This range is considered suitable, as the M(T) anomalies that could be related with phase transformations appear at much higher temperatures (−23°C), as reported in Figures 8 and 9.

**Figure 13.**
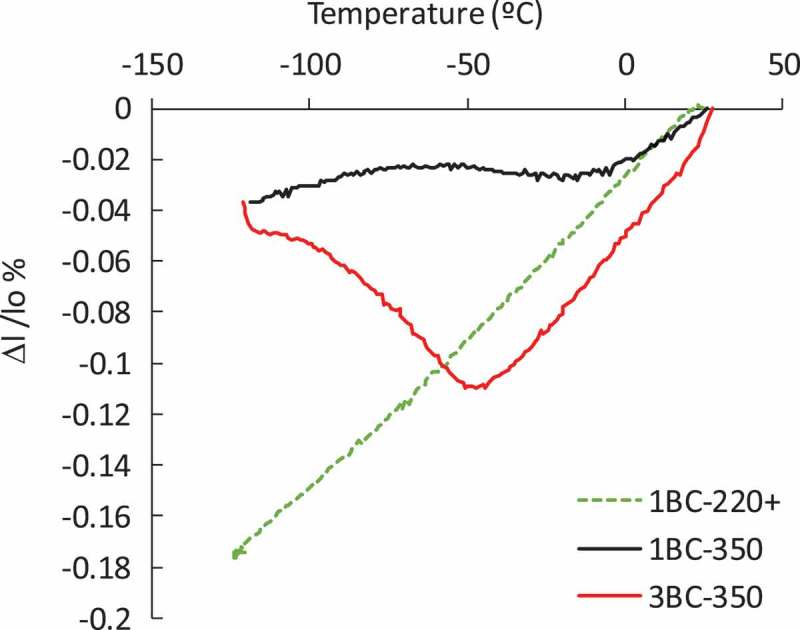
Dilatometric measurements on cooling from RT down to −123°C for samples 1BC-220+, 1BC-350 and 3BC-350. Δl/lo represents the relative change in length.

The dilatometry measurements are shown in Figure 13. There is no evidence of martensitic transformation from retained austenite in the 1BC220+ sample upon cooling from RT to −123°C, as there is no deviation from the expected linear contraction. On the other hand, 350°C bainitic microstructures exhibit deviations from linearity on cooling, indicating the existence of a phase transformation: the decomposition of austenite into martensite leads to a net expansion [68,69]. From these curves, the beginning of the martensitic transformation has been determined to take place at T = −7°C (M_s_) and T = −40°C (M_s_) for 1BC350 and 3BC350, respectively. Note that expansion is larger for 3BC350 than for 1BC350, what agrees with an also higher difference between V_γ_(m_s_1) and V_γ_(m_s_2), as observed in Figure 11.

The transformation detected from dilatometry is barely reflected in the M(T) curve of the 3B350 sample (see Figure 9(b)), where a small anomaly at around −40°C might indicate the start of the martensitic transformation. As the austenite decomposition into martensite is expected to evolve progressively in a wide temperature range, it is not strange that such subtle and progressive transformation might not leave more evident traces on the mentioned magnetic curves (Figure 9(a,b)). Note that for the 1B350 sample, the martensitic transformation starts at higher temperatures (M_s_ = −7°C) than that of the anomaly observed in the M(T) curve (−23°C) (see Figure 8(a)).

Finally, in order to provide further additional experimental evidence, XRD analysis was performed on the three samples that were measured in the dilatometer, to directly check the degree of transformation. These results are gathered in Table 3 and they agree with the previous discussion; i.e., the microstructures of 1B220+ and 1BC220+ are almost identical in terms of V_γ_, which means that no martensitic transformation took place, while in the case of 1BC350 and 3BC350, 9.5 and 10.7 of fresh martensite (V_α’_), respectively, was formed on cooling.

### 3.5. Magnetic behavior versus γ→α’ transformation

Once it has been established that for 1B220+ there is not martensitic transformation down to −123°C, there must be another effect that can explain the detected M(T) increase, below −23°C, when cooling the sample down to the cryogenic regime (see Figure 8).

It is known that the magnetic state of γ (fcc), in Fe and Fe-based alloys, is strongly frustrated at cryogenic temperatures, which leads to the establishment of different magnetic structures, such as ferromagnetic (FM), antiferromagnetic (AF), double layer antiferromagnetic (AFMD), Spin Spiral (SS) …, all of them with close energy values [70–73].

At T = −23°C, the ferromagnetic bainitic ferrite matrix is already ordered, as its Curie temperature is quite far above, ~770°C. As the inset in Figure 8 shows, the change associated with the proposed transition below −23°C it only represents a small, yet detectable, variation of M(T) as compared to that of the initial microstructure, composed of 86% well ordered ferromagnetic ferrite. In other words, such change can only be attributed to a secondary phase, i.e., austenite.

An interesting observation is that the measured austenite microstrain, before and after the dilatometric cryogenic cycle at ~ −123°C, decreases significantly, from 0.0041 down to 0.0029 (%) (see Table 3). This fact points out a microstrain relief associated with the FM ordering of the austenite, as theoretically predicted by Okatov et al. [74]. On the other hand, Boukhvalov et al. [72], by means of *ab initio* electronic structure calculations, have shown that C, as an interstitial present in an Fe fcc lattice (austenite), stabilized a FM state for the Fe atoms at the low temperature ground state (−273°C), making its energy to be lower than that of the antiferromagnetic (AF) state. Therefore, for the particular case of the 1B220+ microstructure, the described microstructural features defining the retained austenite (mainly as thin C-enriched films) seem to lead to its thermal stability when going to temperatures such as low as −123°C, at the same time that a FM state for the Fe atoms is established.

Thus, it is proposed that the mentioned anomaly at −23°C, in Figure 8, is the result of a ferromagnetic transition of austenite, i.e. Curie temperature, T_C_ ~ −23°C.

On the other hand, microstructures such as 1B350 and 3B350, with a higher proportion of austenite with block morphology, and martensitic transformations taking place at −7°C and −40°C, respectively, due to their lower thermal stability, exhibit a sharp M(T) decrease as the temperature decreases below −23°C, where an increase should be expected as a consequence of the appearance of a ferromagnetic phase (fresh martensite α´) and the FM order of the thin films of austenite (see Figure 9(a,b)).

The decrease in M(T) is rationalized in terms of the antiferromagnetism of the untransformed *blocky* austenite, which overcomes the expected increase due to the presence of α´ and thin films. In such *blocky* austenite, much poorer in C than the thin films [23–29] the AF order is energetically favored between those Fe atoms of the austenite cell with less C neighbors [72,75–77]. In the present case, the establishment of such AF order is also found to be around T_N_ = −23°C (Néel temperature). Therefore, the decrease of the M(T) is the net result of the positive FM contribution of α´ and thin films, and the negative contribution of the AF *blocky* austenite. Such overlapping of counteracting effects is evident when considering the smoother decrease of M(T) in 1B350, where M_s_ ~ −7°C > T_N_, as compared to that of 3B350, where M_s_ ~ −40°C < T_N_ (Figure 9(a,b)).

In these particular cases, 1B350 and 3B350, the measured microstrain before and after the cryogenic cycle (RT→ −123°C → RT) at XRD shows an increase from 0.0018 and 0.0016 to 0.0020 and 0.0023 (%), respectively. Such increase would be consistent with the formation of martensite on cooling, and the concomitant introduction of dislocations due to its displacive character. In these samples, the microstrain does not seem to change because of the establishment of the magnetic order (AF).

An intermediate scenario, where the calculated ratio between thin films and blocks of austenite is around 1, and no martensitic transformation was detected on cooling down to −123°C, corresponds to the sample denoted as 3B250 (see Table 3 and Figure 3). The thermal variation of this sample, presented in Figure 9(c), shows a pronounced cusp centered around −23°C. This scenario reflects again the competition between the FM arising from the bainite and thin films of austenite (prevailing at T > −23°C), and the AF of the blocks (prevailing at T < −23°C).

It is clear now that the irreversibility observed in the M(T) curves for all the analyzed samples can not be attributed to a martensitic transformation of the retained austenite, opposite to what occurs in a Cr-alloyed high C steel cooled down to −200°C, as reported by Tavares et al. [46]. Following, this feature will be explained in terms of magnetism.

There is a well known magnetic proximity effect that may propagate the spin polarization of a magnetic metal into a nonmagnetic one, as first reported by Hauser in 1969 [78]. Since then, several works have used this effect to explain induced magnetic behaviors. Unguris et al. [79] reported a spin-density wave (SDW) antiferromagnetism in Cr films deposited on an Fe substrate, at temperatures above the T_N_ of bulk Cr, due to the coupling of Cr and Fe at some distance from the interface. Maccherozzi et al. [80] reported a magnetic coupling between Mn and Fe in (Ga, Mn) As/Fe interfaces that extends over a region as thick as 2 nm. Kravets et al. [81] investigated the interlayer exchange coupling in a ferromagnetic Ni-Cu trilayer system (strong/weak/strong) and explained the paramagnetic-to-ferromagnetic transition of the spacer material by the magnetic proximity effect. Furthermore, Zhang et al. [82] reported the application of this effect to their *ab initio* simulations of structural transformations between austenite and ferrite in Fe-C alloys. And such a strongly coupling between both phases supported also the work of Razumov et al. [83] about phase transformations in steels. In the same line, the actual work proposes that this magnetic proximity effect is at the origin of the low-T magnetic order hold in austenite when heating to RT and, thus, explains the observed irreversibility in the thermal evolution of the magnetization. Zhang et al. [82] reported that the exchange parameter, J, of FM bcc Fe (~0.2eV) is much larger than that of the paramagnetic (PM) fcc Fe (close to 0eV), so that the Fe atoms at the interface between PM austenite and FM ferrite are more strongly coupled to the FM ferrite side, and thus more likely to follow the FM ordering. In this sense, for the samples studied in the present work, as the bainitic ferrite surrounding the retained austenite (both *film-type* and *blocky-type* in the different samples) is FM ordered down from ~770°C a ferromagnetic coupling to Fe atoms of the austenite films at the interface between both phases can be expected. Such Fe atoms will be AF or FM ordered depending on the microstructural and chemical factors relative to each sample, as already discussed, with a T_C_ or T_N_ around −23°C, and above this temperature they are PM. However, the exchange parameter, J, for FM bainitic ferrite is supposed to be larger than that for the PM austenite and, thus, it can keep the magnetic order already established at low temperatures.

## 4. Conclusions

In the present study, bainitic microstructures consisting of ferrite plates of nano- or submicro-sized dimensions interspersed with thin-films of C-enriched retained austenite, and *blocky* austenite, were produced by isothermal treatment at different temperatures of three high C and high Si steels.

Their magnetic properties have been characterized and correlated with very particular and distinctive microstructural features. The study has been performed at both, room temperature and in the cryogenic range. The main findings and conclusions are:

- At cryogenic temperatures, retained austenite thermal stability was characterized by the occurrence, or not, of martensitic transformation. In those microstructures where the morphology of retained austenite is predominantly thin-film, martensitic transformation did not occur when cooling down to −123°C; other microstructural factors such as microstrain, scale of the matrix or austenite carbon content have been found to also play an important role. A microstructural stability parameter (msp) that accounts for almost all the mentioned factors influencing the stability of austenite is proposed.
- Thermomagnetic measurements have brought significant differences on the magnetic behavior of the studied samples to light. Thus, a ferromagnetic order is detected for microstructures with a higher thin film/blocky ratio (prevalence of *film* type austenite), while an antiferromagnetic order is exhibited by those with lower film/blocky ratio (prevalence of *blocky* type austenite). In fact, a competition of both magnetic behaviors has been found for all the studied samples, always showing the transition temperature around −23°C, transition that has been attributed to the retained austenite.
- Furthermore, these magnetic transitions of austenite are irreversible for all the samples, suggesting a proximity magnetic effect between such phase and the ferromagnetic ones (T_c_~770°C) surrounding it.
